# Participant Outcomes and Facilitator Experiences Following a Community Living Skills Program for Adult Mental Health Consumers

**DOI:** 10.1007/s10597-022-01020-x

**Published:** 2022-09-08

**Authors:** Eliza Sammells, Alexandra Logan, Loretta Sheppard

**Affiliations:** 1 Occupational Therapist, BOccThy (HONS), Australian Catholic University, Melbourne, Australia; 2 Occupational Therapy Senior Lecturer, School of Allied Health, Faculty of Health Science, Australian Catholic University, Melbourne, Australia; 3 Occupational Therapy, Deputy Head, School of Allied Health, Australian Catholic University, Melbourne, Australia

**Keywords:** Occupational therapy, Living skills, Adult mental health, Recovery, Homelessness

## Abstract

Outcomes from participating in a six-week small-group living skills program, periodically implemented over two years with twelve adult mental health consumers, are reported as a case study. Occupational therapy and recovery-oriented approaches underpinned the development and implementation of the program thus it was important that outcome measures aligned with these perspectives. Data were gathered pre and post program using the Canadian Occupational Performance Measure (COPM) and the Life Skills Profile-16 (LSP-16) in each iteration of the program. Two occupational therapy program facilitators were interviewed about their experience of running the program and using these measurement tools. COPM occupational performance (*p* = 0.001) and COPM satisfaction (*p* = 0.004) scores indicated significant change at post-program whereas the LSP-16 total and subscale scores did not *(p = 0.132 for total scores)*. Facilitator insights into the experience of implementing the living skills program and the perceived alignment of the COPM and LSP-16 with recovery-oriented practice are reported.

## Introduction

Mental illness is a leading global health concern (Australian Institute of Health and Welfare, 2018). The complex and dynamic impact of symptoms, stigma and access to supportive treatments can impede a person’s ability to meet daily needs and perform optimally (Caplan, 2014; D’Amico et al., 2018; Gibson et al., 2011). Recovery reform is shifting the mental health sector from a ‘symptom focused’ approach to a model of care that values consumers decision making and control over their life (Jacob, 2015). Recovery is defined in the Australian national framework for mental health services as ‘being able to create and live a meaningful and contributing life in a community of choice with or without the presence of mental health issues’ (Australian Government Department of Health, 2013, p. 2). The Australian policy shift reflects the growing body of literature and evidence to support recovery-oriented practice as an alternative to interventions that focus on symptom reduction. Davidson (2009) identified nine ‘elements’ that support health professionals to adopt a recovery-oriented approach to practice: involvement in meaningful activities, redefining self, managing symptoms, empowerment and citizenship, assuming control, overcoming stigma, renewing hope and commitment, being supported by others, incorporating illness and belonging.

Occupational therapists are key members of the mental health workforce. The profession’s core values reflect and align with recovery-oriented practice, and evidence affirms participation in meaningful activities and occupations as a pathway to facilitating recovery, health and wellbeing (Davidson, 2009; Baker & Procter, 2014; Townsend & Polatajko, 2013). Living skills are those skills necessary or desirable to meet the needs of daily life (Davis & Kutter, 1998), for example financial management (Caplan, 2014; Rosen et al., 2009), home management (Gibson et al., 2011) and nutritional meal preparation (Carson et al., 2013) which help form the basis for a ‘meaningful and contributing life’ as per the Australian Government definition of recovery (Australian Government Department of Health, 2013, p. 2). Occupational therapy living skills programs are aimed at increasing participation in meaningful activities, creating opportunities to experience some of the nine recovery elements to promote a path to recovery for mental health consumers (Aviles & Helfrich, 2006; Davidson, 2009; Helfrich et al., 2012). Evidence also indicates that participating in living skills programs can improve quality of life (Eftimovska-Tashkovska et al., 2016; Gibson et al., 2011; Svedberg et al., 2014), reduce mental health symptoms (Chen et al., 2015; Katz & Keren, 2011) and enhance occupational performance (Helfrich et al., 2012; Štrkalj-Ivezić et al., 2013; Tungpunkom et al., 2012) but there remains a challenge in measuring outcomes from living skills programs based on recovery-oriented practice that capture both the effect of living skills programs and the link to recovery (Barbic, 2017).

Recovery implies change and is complex, dynamic and multifactorial (Davidson, 2009), and living skills programs that are individualised, goal oriented and incorporate participant choice have the greatest positive effect on participants (Helfrich & Chan, 2012; Lindström et al., 2012; Svedberg et al., 2014). The simultaneous use of recovery-oriented practice and theoretical underpinnings from occupational therapy holds promise as a way to conceptualize and measure outcomes from participating in a living skills program aligned to recovery. However, the applicability of tools commonly used in community mental health practice is uncertain and warrants investigation.

Commonly used outcome measures in occupational therapy community mental health practice include the Canadian Occupational Performance Measure (COPM) which was developed from a well-established occupational therapy framework, the Canadian Model of Occupational Performance and Engagement (CMOP-E). This framework considers the interplay between person, environment and occupation and the resultant impact on occupational performance (Townsend & Polatajko, 2013). The COPM is a person centred, strengths focused tool with robust metrics designed to measure change in participant self-rated performance in everyday activities and their satisfaction with that performance (Law et al., 2014). It aligns with recovery-oriented practice as described by Davidson (2009) by emphasising choice, control and involvement in meaningful activities (Law et al., 2014) but does not measure recovery per se. A more generic mental health tool, the Life Skills Profile − 16, is a commonly used measure of living skills function in the Australian community mental health sector. It reportedly offers a clinical and objective assessment of general functioning in basic living skills (Rosen et al., 2006) but it’s alignment to recovery-oriented practice is unclear.

The aims of this study were to (i) explore outcomes following participation in a living skills program for adult mental health consumers using the COPM and LSP-16; and (ii) capture the experiences of the program facilitators, including their perspective on the alignment of these tools with recovery-oriented practice.

## Program Description

The need for a living skills program for adult mental health consumers at risk of homelessness was identified by a community service agency in regional Victoria, Australia. The agency services were guided by a strengths-based, recovery-oriented approach thus the living skills program needed to adopt individualised and recovery-oriented practices in its design, delivery and evaluation. Undergraduate occupational therapy students developed the living skills program in collaboration with agency personnel and professional practice educators to ensure alignment with the agency values. The program was piloted in 2017 with a small group of young adults who were living in supported community housing and evaluated using the COPM and LSP-16. The overall goal of the pilot program was to increase participants’ independence in everyday living skills and participation in the community. Participants undertook activities designed to develop skills in cooking, nutrition, financial planning, budgeting, work readiness and social interaction. The weekly program content which ran for six weeks was designed and structured around participant identified living skills goals with a focus on building on existing strengths and applying developed skills in real-world settings. The group program was reviewed after the first implementation and subsequent programs were supplemented by individual sessions addressing specific participant goals such as supermarket shopping and accessing community venues such as the local library. This enabled the consolidation of skills learned in the group setting for individuals seeking experience in applying their skills in a real-world context. In 2019, the program was expanded to include adults who had already experienced homelessness but were now living in supported community housing.

For all participants, the COPM interview set COPM performance and satisfaction goals related to everyday living and was instrumental in shaping the individualised content of each program. Prior to commencing the program, consenting participants met with the program facilitator, an occupational therapist or occupational therapy student, to undertake the COPM interview and set their individual goals. Progress made towards achieving these goals by the end of the program was used as an outcome measure. Agency staff familiar with the participants completed the LSP-16. The program content was structured around the living skills goals common to a small group (four-six in each program) that could reasonably be undertaken in the group setting twice per week over a six-week period supplemented by individual sessions where necessary and possible. Common occupational domains addressed through the program included shopping, meal preparation and budgeting.

## Research Questions

The first research questions focused on individual outcomes for participants of the Living Skills program: What change occurred following participation in the program with regard to (i) participant occupational performance and satisfaction as measured by the Canadian Occupational Performance Measure (COPM), and (ii) participant general functioning as measured by the Life Skills Profile-16 (LSP-16)?

The second group of research questions explored the experience of the occupational therapists running the program, specifically seeking to understand their perspective on (i) facilitating the program, (ii) the alignment of the program with recovery-oriented practice and (iii) the suitability of the outcome measures to capture change and align with recovery-oriented practice.

## Methods

Given the contemporary “real world” context of the living skills program, a case study approach to reporting the findings was considered most appropriate. Data were gathered from multiple sources: participant self-ratings via the COPM, support worker ratings via the LSP-16, and experiential reports via program facilitator interviews. Multiple sources of data collection converging in a triangulating manner are well suited to real-world evaluations (Yin, 2018; Jones & Lyons, 2004). COMP performance and satisfaction scores were collected prior to and following participation in the living skills program, as were the LSP-16 ratings. Semi-structured interviews with the two occupational therapy facilitators explored their perceptions of the program’s alignment with occupational therapy mental health and recovery-oriented practices, including the alignment of the measurement tools. A reflexive thematic analysis (Braun & Clarke, 2022) was used to analyse the transcripts from these two datasets in keeping with the flexibility in dataset size and composition that this analytic method offers.

Ethical approval to conduct the research was provided by the Human Research Ethics Committee [2017-335 H]. Data collection occurred intermittently from November 2017 to August 2020.

### Living Skills Program Participants

Convenience sampling was used to recruit participants from adult consumers at risk of homelessness residing in supported accommodation provided by a community mental health service in regional Victoria. Inclusion criteria required participants to be adult consumers of the organisation with identified needs for support in everyday living who were willing to be referred to the program by their case manager. To maintain a safe environment, participants were unable to attend group activities if they were under the influence of drugs or alcohol. No other exclusion criteria were applied. Information letters and consent forms were distributed via the service coordinators and case managers. Although individuals were able to participate in the living skills program without participating in the research, the same evaluation processes were undertaken as part of the program implementation. Written informed consent was obtained and measurement tools and data analysis processes were structured to enable enhanced understanding of participant results and the experience of program facilitators. All participants of the program consented to have their data included in the research project but three did not complete the program.

### Measurement Tools

*The Canadian Occupational Performance Measure (COPM).* The COPM is an occupation-based goal setting tool that can be used pre and post intervention to measure change in occupational performance and occupational satisfaction (Canadian Ocupational Performance Measure, 2020; Law et al., 2014). The tool is responsive to change and has good clinical utility, taking fifteen-thirty minutes to administer (Law et al., 2014). The COPM is administered by an occupational therapist (or occupational therapy student) in a semi-structured interview aimed at identifying and prioritising participant goals. The participant ranks their perceived performance and satisfaction from ‘can’t do it at all’ or ‘not satisfied at all’ (1) to ‘can do it extremely well’ or ‘extremely satisfied’ (5). The COPM has been found reliable (r = 0.84) (Pan et al., 2003) and deemed an effective tool for adults living with mental illness (Carswell et al., 2004; Parker & Sykes, 2006). A change of two points or more implies a clinically significant change (Law et al., 2014).

*The Life Skills Profile (LSP-16).* The abbreviated form of the Life Skills Profile (Rosen et al., 2006), the LSP-16, is a commonly used measurement tool in community mental health in Australia. It is completed by a case manager or mental health practitioner familiar with the consumer to provide a clinical and objective assessment relating to the person’s general functioning in basic living skills (Rosen et al., 2006). The LSP-16 was derived from the more extensive earlier version of the Life Skills Profile and includes four subscales: self-care, anti-social, withdrawal, compliance. The practitioner ranks the occurrence of itemised objective behaviours from ‘not at all’ (0) to ‘often’ (3), where a higher score indicates a lower level of function. Subscale scores are calculated by totalling individual item scores. The Life Skills Profile and derived versions have demonstrated adequate internal consistency (Eagar et al., 2005), re-test reliability and concurrent validity (Trauer et al., 1995). The LSP-16 is not designed to be re-administered within a three-month period (Rosen et al., 2006) so post-program data were collected at least three months following the pre-program data collection.

### Quantitative Data Collection

Data were collected from programs running between November 2017 and August 2020 with small data sets from successive programs forming the total data set in this study (see Table 1 for an outline of successive data collection). The pilot program targeted meal preparation and cooking skills. The COPM and LSP-16 were used in the pilot program, but some LSP-16 data sets were incomplete and therefore excluded from the data analysis. In alignment with participant goals, three programs targeted meal preparation and cooking skills and one program targeted budgeting and financial management. The duration and delivery of the program was adapted to meet the needs and goals of the participants on each occasion.

**Table 1:** Data Collection

Year	Number of participants	Measurement tools	Program delivery mode
2017^a^	4	COPM, LSP-16	5 weeks, weekly group and individual session.
2019	5	COPM, LSP-16	6 weeks, weekly group session.
2019	5	COPM, LSP-16	5 weeks, weekly individual and group session.
2020	3	COPM, LSP-16	3 weeks, two group sessions per week.
^a^Pilot program			

### Quantitative Data Analysis

Descriptive statistics using SPPS Version 26 (IBM, 2019) were used to represent quantitative data. Normal distribution was not assumed, therefore non-parametric analyses were undertaken. A Wilcoxon signed-ranked test was used to conduct group analysis between two time points at pre-program and post-program for COPM and LSP-16, ensuring that the required three-month time period had elapsed by post-program administration for the LSP-16. The significance level was set at p < 0.05.

### Living Skills Program Facilitator Participants (Occupational Therapists)

Purposive sampling via email was used to recruit occupational therapists or occupational therapy students who had facilitated the living skills program in its entirety at the rural community mental health organisation and were willing to participate in a semi-structured interview. Access to facilitators from earlier years was limited, however two facilitators from a total pool of five consented to be interviewed. Information letters were provided. One occupational therapy program facilitator was also involved in some of the data collection for the quantitative strand of this study, presenting a potential risk of bias. No other conflicts of interest were noted.

### Semi-Structured Interviews

Semi-structured interviews via tele-conference were used to explore the experience of facilitating the living skills program, including facilitator perception of the program’s alignment with recovery-oriented practice and the suitability of the measurement tools. The dataset size and composition aligned with the flexibility available when using reflexive thematic analysis as proposed by Braun & Clarke (2022). One interview was 24 min and the other was 45 min long and given the two participants constituted 40% of the total available pool, the interpretation of their story was considered valid and worthwhile to add context and meaning to the quantitative datasets. Guiding interview questions included:

- What was your experience of facilitating the living skills program?
- How did the program and measurement tools align with a recovery-oriented approach?
- Did the measurement tools capture changes you observed or noticed over the course of the program?

The data were transcribed verbatim, and the transcription compared with the recording to ensure accuracy. Familiarity with the data was achieved by listening to the transcripts several times, making brief notes and reading and re-reading the transcriptions (Braun & Clarke, 2022).

### Thematic Analysis

Critical and inductively orientated analysis based on principles of thematic analysis (Braun & Clark, 2019) was used to identify, analyse and report shared patterns of meaning from the two semi-structured interviews. The researchers brought an occupational therapy and recovery-oriented practice lens to this process and were guided by the six phases outlined in Braun & Clarke (2006) and in their subsequent publications (2019 and 2022): (i). Familiarize, (ii) Generate initial codes, (iii) Generate themes, (iv) Review themes, (v) Define and name themes, (vi) Produce the report. Within the constraints of limited transcription data from two participants, codes and themes were generated by the first author according to shared meaning. All three authors reviewed the data before finalising themes through comprehensive thoughtful discussion and reflection. Potential for internal bias was monitored and managed through a reflexive journal (Nowell et al., 2017). Participants were emailed a summary of the themes to ensure accuracy and enhance credibility.

## Results

### Quantitative Results

Participants were those living with various mental health conditions, for example: experiences of trauma, schizophrenia, alcohol and other drug use, depression, anxiety and eating disorder. Seventeen datasets were eligible for inclusion in the analysis (see Table 1). Three people did not complete the program and their data were removed from the dataset. The 14 remaining datasets were drawn from 12 people, four of whom participated in the program twice and whose data were treated as separate datasets, one set per program. Two datasets were incomplete in COPM data, and three were incomplete in LSP-16 data. These data were therefore removed leaving 14 COPM and 13 LSP-16 datasets for analysis (*see* Table 2). The final participant sample included four females and eight males aged between 17 and 50 years (mean = 31.9 years).

**Table 2:** Pre-Intervention/Post Intervention Results

Measurement tool Domain	*n*	Pre-intervention Median (interquartile range)	Post-intervention Median (interquartile range)	z-score	p value
COPM	14				
Occupational Performance		3.8 (1.5)	5.8 (2.0)	− 3.297	0.001*
Occupational Satisfaction		3.9 (1.8)	6.0 (2.2)	-2.905	0.004*
LSP	13	19.0 (5.0)	17.0 (8.0)	-1.505	0.132
Withdrawal		5.0 (4.0)	4.0 (5.0)	− 0.889	0.374
Self-Care		8.0 (4.0)	8.0 (3.0)	− 0.627	0.531
Compliance		3.0 (1.0)	2.0 (2.0)	− 0.552	0.581
Antisocial		5.0 (2.0)	4.0 (3.0)	-1.293	0.196
*Significant at p < 0.05					

**Fig. 1:**
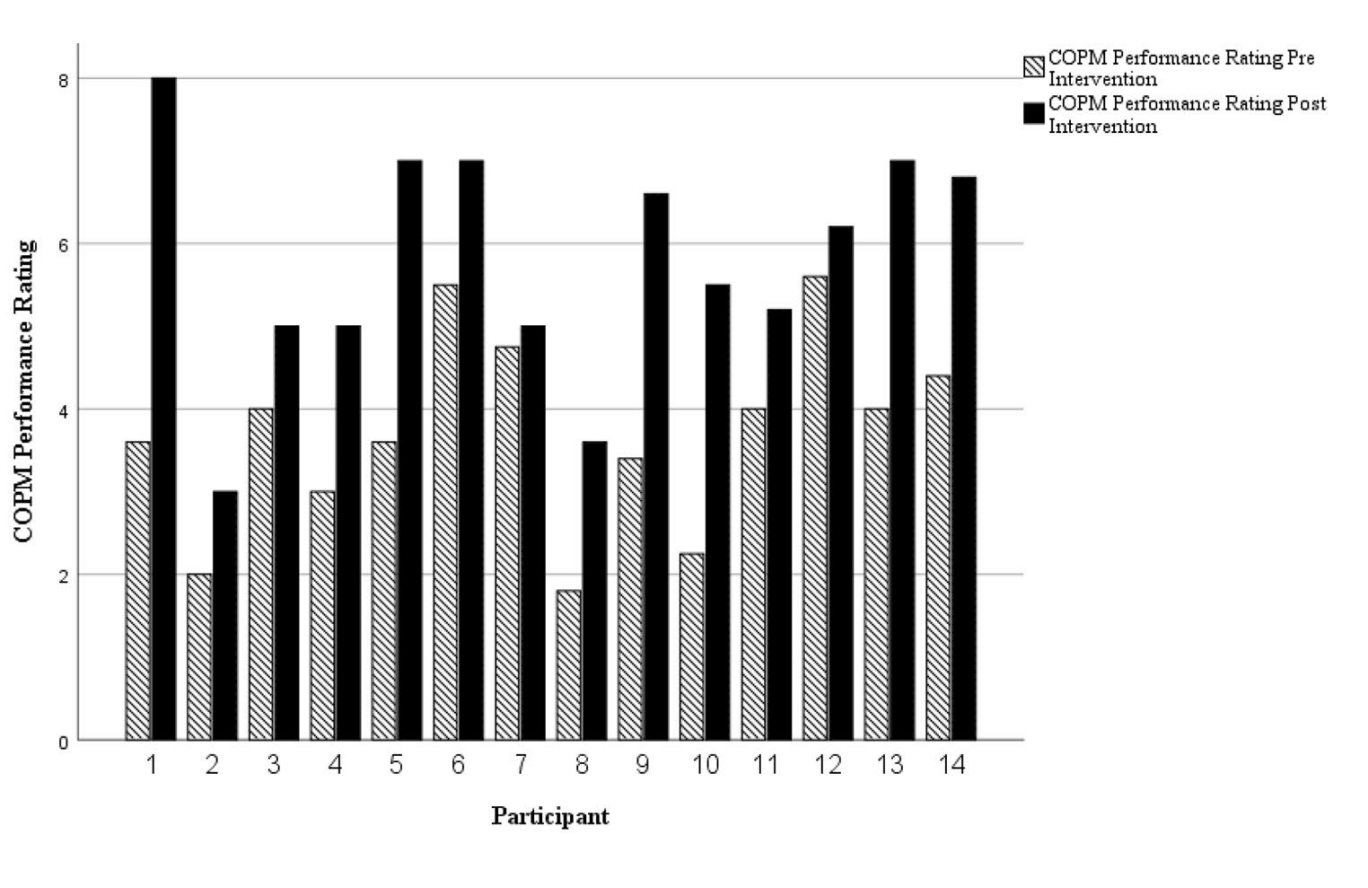
Individual Occupational Performance Case Analysis as measured by the COPM

**Fig. 2:**
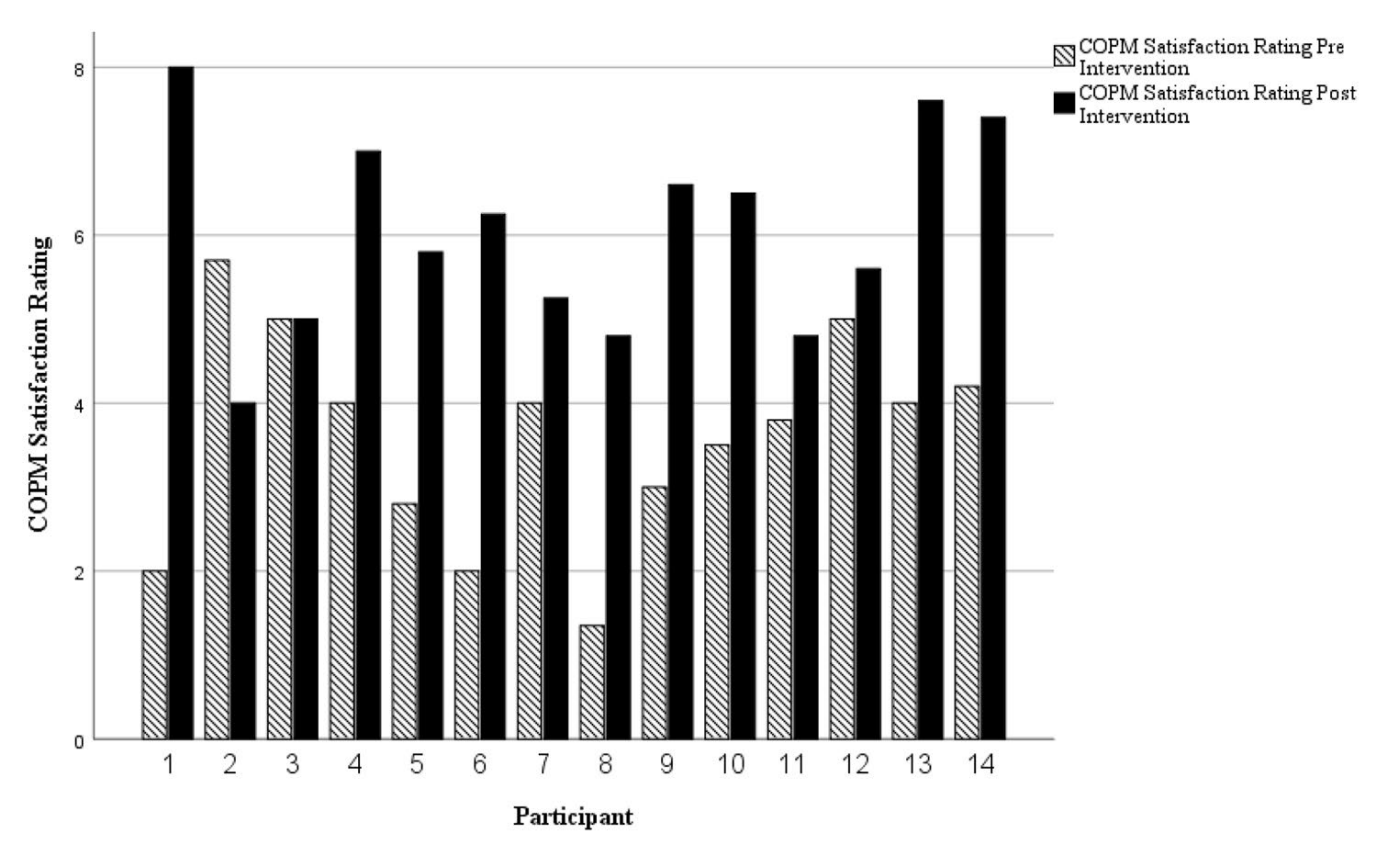
Individual Occupational Satisfaction Case Analysis as measured by the COPM

COPM scores in occupational performance *(z = -3.297, p = 0.001)* and satisfaction *(z = -2.905, p = 0.004)* significantly improved from pre-intervention to post-intervention. Individual case analysis outlining occupational performance is presented in Fig. 1. and occupational satisfaction in Fig. 2.

The LSP-16 showed no significant change in the total scores *(z = -1.505, p = 0.132)*, nor across any domains from pre intervention to three-months post intervention *(see* Table 2*).* The LSP-16 was completed by the same mental health practitioner pre and post program for each individual participant.

### Thematic Analysis

One male and one female occupational therapist participated in the semi-structured interviews. One facilitator, pseudonym MS, was an occupational therapist with two years of service with the organisation. The other facilitator, pseudonym NP, was an occupational therapy student when implementing the program in 2019 and had since begun working as a registered occupational therapist outside of the organisation.

**Fig. 3:**
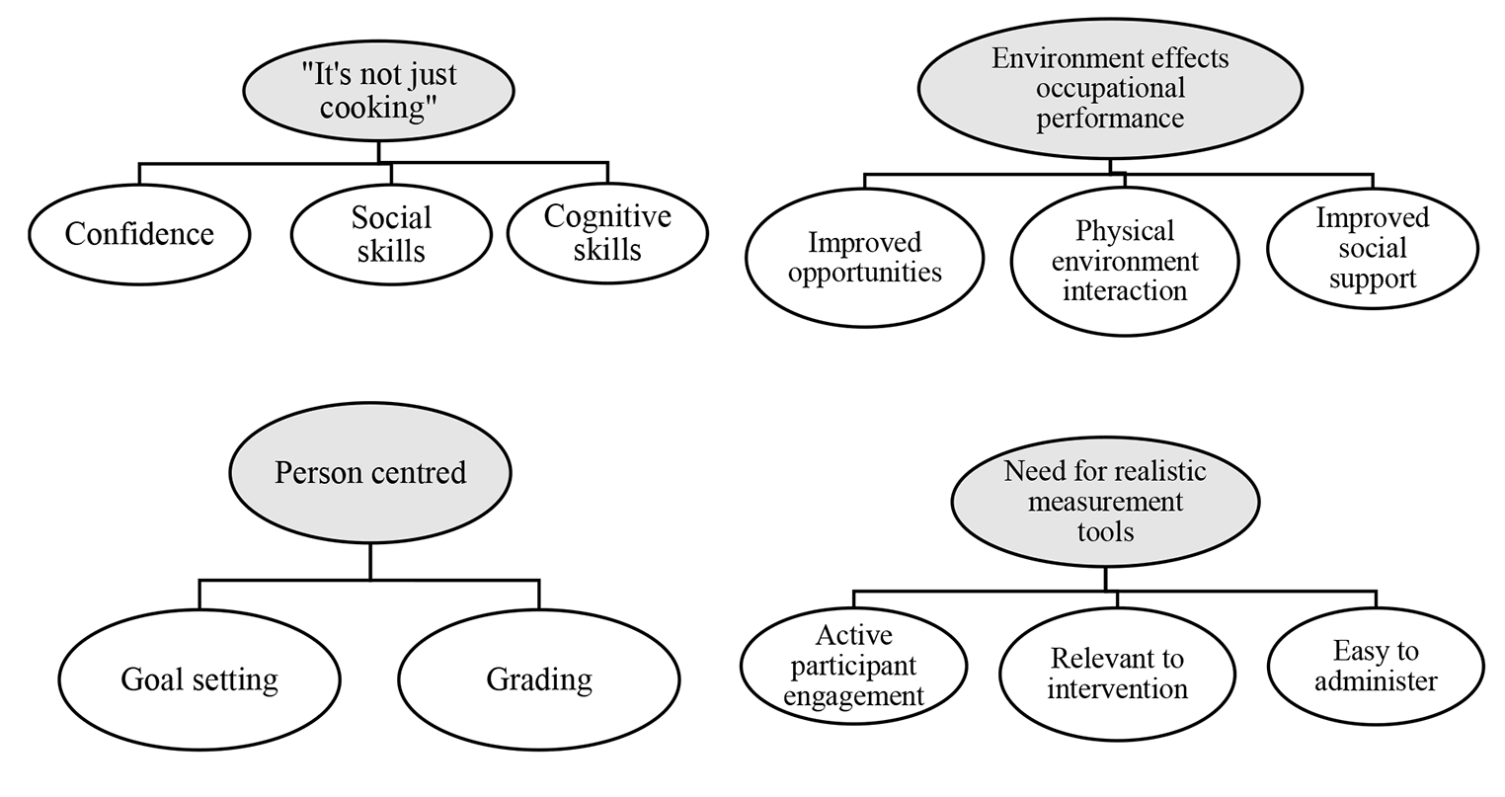
Theme network

Four themes were generated: *‘it’s not just cooking’*, ‘*environment affects occupational performance’*, ‘*person centeredness’* and ‘*a need for realistic measurement tools’*. Written informed consent was obtained from participants to include direct quotes. Figure 3. presents a theme network summary.

### Theme 1: “It’s Not Just Cooking”

Facilitators explained that a range of skills were developed through participation in the program. Cooking was outlined as a therapeutic and complex task with “a whole other set of skills underneath”. Skills developed in the program were seen as being able to be “transferred” into other settings and areas of life.

Cognitive skills such as “planning, delegating and problem solving” were reported to consequently develop through participation. Such skills often complimented other participant goals such employment and organisational skills. The program occurred at a set time each week which provided structure and “routine”. NP expressed that some participants “struggled with getting to an appointment”. Thus, the program provided opportunities for development of organisational skills.

Opportunities for “leadership roles”, “teaching others” and “conflict resolution” were reported. Social interaction is embedded in the program through group participation and provides a safe space to develop social skills. Furthermore, facilitators expressed that “confidence” was a personal attribute that developed through participation in the program. NP described participants as “proud” and noticed an increased motivation to cook outside of the program: “one (participant) went away and cooked her own meal……another went to the library and printed out a bunch of recipes”.

Facilitators emphasised that participating in the program is much more than “just cooking” or budgeting. NP reported that the program emphasised personal development and ‘sense of self’, explaining that “you’re not just doing sessions for the sake of it being another week, you’re more so doing them because you’re going onto the next step.” He reported to challenge participants with questions such as “where do you want to go next, what’s the next step for you?”. He elaborated “it allows them to continue to develop to areas they wouldn’t have outlined in their goals”, “because it’s a confidence thing, it’s about going into those environments that would potentially trigger you and coming out with not only a skillset, but the confidence and the motivation to continue on with what the programme’s taught you.”

### Theme 2: Environment Affects Occupational Performance

Facilitators expressed the significant impact of the environment for consumers. The environment was conceptualised through an occupational therapy CMOP-E lens whereby the ‘environment’ is the context in which an individual performs occupations and includes physical, social, cultural and institutional elements (Townsend & Polatajko, 2013). The facilitators emphasised that most consumers were transitioning “from one extreme environment to another, from living on the streets to going into housing”. They described that the physical environment of living homeless denied and prevented opportunities for participation in everyday living activities. Consumers did not “have the ability to engage in those occupations such as self-care and productivity in particular”. The program encourages and facilitates functional interaction with the physical environment. MS reported that “at the start of the program they’re not interacting with their environment, they’re getting takeout … whereas by putting them in the program … they’re able to progress to the point where it’s independent”.

The facilitators also expressed that the social environment affects opportunities for participation. The facilitators explained that “a lot of these guys have spent the majority of their life [living] independently, lack of family support and lack of social supports”. Group sessions provided opportunities for peer mediation, role formation and “peer support”. MS explained that a participant who had a substantial baseline skillset changed his goals and reported “I’ve obviously got this knowledge and I’ve got a good skillset, how can I then transfer that to other participants to help them improve as well”. Peer mediation provided beneficial opportunities for all parties involved.

Facilitators explained that the program is held within a “good space of support”. Participants in the 2019 and 2020 programs had access to support from staff from 9am-5pm. Participants also work with assigned case managers so that goals that were not addressed in the program could be worked towards outside the program. The facilitators expressed that the level of support and physical environment “complements” the program and enhanced opportunities for occupational performance and satisfaction and improved quality of life.

### Theme 3: Person Centred

Person centeredness was expressed as a “foundational principle” of the program. MS outlined “if it’s not person centred … it’s not really going to benefit … they’re not going to engage and they’re not going to come back next week”. The COPM was used as a pivotal tool that directed the program towards involvement in individualised meaningful activities. MS described “it’s about what they’re wanting to get out of the program, from their point of view”. MS reported that the program is “providing support in a really crucial time” where there were realistic opportunities for participants. The intervention was matched to the participants current life opportunity.

The facilitators placed emphasis on “follow up” and documenting weekly objectives with participants. This construct was described to participants by, “you’ve done this this week, where do you want to go next, what’s the next step for you?” The program was adapted by the facilitator to support individual goal attainment, group dynamics and continual development. Grading by increasing or decreasing the level of difficulty was used to adapt activities to promote improved occupational performance, for example, to develop skills through selection of simple to more complex recipes, but also within role competencies. Participants were provided with opportunities to fulfill roles with increased accountability and responsibility within the group. For example, some participants volunteered to assist in selecting recipes, other participants became peer mentors.

### Theme 4: Need for Realistic Measurement Tools

Facilitators stated that measurement tools needed to be relevant, easy to administer and engage the participant. The COPM was championed due to active participant engagement. The LSP was not strongly endorsed due to a lack of participant involvement and limited change captured.

Facilitators noted that the measurement tools need to be relevant to the intervention. They suggested that the inclusion of the COPM was valuable, however it is a broad tool and change may not be reflected within the program timeframe of 3–6 weeks. MS stated that “less than 3 months doesn’t really give you enough time to work on their five established goals”. The facilitators also acknowledged the impact of confounding variables on measurement tools such as: stable housing, increased support, symptom management and daily life occurrences.

Facilitators emphasized that active participant involvement, the usability and relevance of the measurement tool determines its value in relation to measuring meaningful change and were strengths of the COPM.

## Discussion

This study is reported using case study methodology which allows findings to be drawn from a range of sources where the context is specific and sample sizes small (Yin, 2018). The study provided the opportunity to explore outcomes for participants of the program, and experiences of facilitators in using recovery-oriented practice combined with an occupational therapy living skill program. Results support the use of both a recovery-oriented, and an occupational therapy approach. Analysis of the facilitator interviews suggest participants made gains in areas related to Davidson’s recovery elements, specifically those elements of involvement in meaningful activities, redefining self, assuming control, being supported by others, and belonging. COPM results indicated good progress towards identified goals, and this aligns with Davidson’s assuming control element and involvement in meaningful activity. LSP-16 data indicated no change and raises questions about the tool’s sensitivity to change. Facilitator interviews also raised questions about the alignment of the tool to recovery-oriented occupational therapy practice as it was neither person-centred nor participant self-report and appeared distant to any of Davidson’s recovery elements.

Involvement in meaningful activities as an element of recovery was embedded in the program through its occupation-based nature and the inclusion of individual goal setting. Outcomes reported in this paper support the role of the COPM in promoting and measuring involvement in meaningful activities. This is consistent with previous evidence demonstrating effective goal setting has positive effects on personal recovery and self-efficacy (Alanko et al., 2019; Rose & Smith, 2018) and that personalised goal setting and goal facilitation is a pathway to recovery (Kirsh et al., 2019).

Although interpretation is limited by the small dataset, the facilitator interviews provided insight into the experience of implementing the living skills program using personalised goal setting and goal facilitation, and perspective on whether this aligned with a recovery-oriented approach. It appears that the COPM provided the opportunity for participants to enact choice and control. This is consistent with the ‘assuming control’ element of Davidson’s recovery model (2009) which situates participants as experts in their own experience and encourages them to guide the approach to their own support needs. Program facilitators reported a perceived increase in participant confidence, self-determination and self-efficacy over the duration of the living skills aligning somewhat with the ‘redefining self’ element of Davidson’s model.

Facilitator interviews also indicated an increase in living skills program participants’ self-confidence and self-worth. The group nature of the program provided participants with opportunities for peer interaction, mentorship and leadership and participants regularly referred to ‘being supported by others’. Eftimovska-Tashkovska et al., (2016) found a group environment also contributed to improved social opportunities, increased motivation and provided a sense of comfort and belonging. Future studies might consider quantitative measurement of these attributes to contribute to our understanding of the impact of participating in living skills programs. Slade (2009) described a conceptual overlap between recovery-oriented practice and quality of life and the World Health Organisation Quality of Life-Brief version (WHOQOL-BREF) may be an appropriate recovery oriented self-administered tool (WHOQOL Group, 1996) worth exploring in future studies.

It is difficult to attribute change solely to the living skills program, partly because of changes to life circumstances experienced by some participants during the program, reflecting the complexity of evaluating ‘real-world’ programs, and partly due to broad and holistic measurement tools such as the LSP-16 which may not be sensitive to change in this context. Recovery is by its nature idiosyncratic, and measurement tools which capture change in elements indicative of recovery are necessary if we are to understand the process of recovery from an everyday perspective. In this study, the program facilitators also noted a positive impact on some participants when they entered the organisation and began to access services simply because of this change in their life circumstances. Several participants transitioned from being homeless to housed at the same time as they participated in the living skills program, with corresponding improved access to social and physical supports in addition to the specific opportunities the living skills program afforded them. Henwood et al., (2015) noted that ‘until an individual has basic safety and security needs met, they will not have an adequate platform to successfully address other challenges’, and it seems reasonable to assume that housing is an important prerequisite for indicators of recovery, including skills in everyday living.

The consumer movement has created momentum within the mental health sector and places emphasis on a recovery-oriented service focus and a strong consumer voice. Andresen et al., (2010) acknowledges the individualistic nature of recovery-oriented approaches and asserts a need for the consumer voice to be heard through self-report or subjective measurement tools, thus individualised goal attainment-based measures, such as the COPM, may be beneficial in supporting recovery approaches (Clarke et al., 2009; Kirsh & Cockburn, 2009) due to active participant involvement through self-report honours the participants expertise in their own experience.

There are limitations to this study. The research was conducted at one organisation across two sites with a total of 12 participants, with relatively small datasets for the two measurement tools used. Having only two participants for the facilitator interviews limited the breadth and depth of analysis and although using principles of thematic analysis which allows for flexibility of dataset size and composition (Braun & Clarke, 2022), this small sample size reduced confirmability and therefore generalisability of findings. Further, the organization provided additional supports for participants concurrent with their involvement in the program making it difficult to attribute findings solely to the living skills program.

Nevertheless, the findings indicate that a living skills intervention that is individualised and incorporates participant choice and group interaction is able to support people to achieve their goals. The results suggest that self-report measurement tools which actively engage participants such as the COPM, are able to demonstrate meaningful change. The findings invite occupational therapists and other allied health professionals to consider the applicability and relevance of measurement tools for recovery-oriented programs conducted in real-life community settings, such as the living skills program.

Recovery is complex and multifaceted and influenced by more than one support or intervention, thus the importance of identifying measurement tools most closely aligned to recovery-oriented practice is paramount. It is noteworthy that the tool most closely aligned to the intervention in this instance, the COPM, was also the tool that demonstrated significant change across the participant group. The holistic, individualised nature of recovery-oriented practice has synergies with occupational therapy in the mental health sector (Kelly et al., 2010) and this study provides preliminary evidence for the effectiveness of living skills programs to improve occupational performance and support recovery-oriented practice with adults living with mental health conditions.
